# Bilateral refractory pneumothorax treated by pleurodesis and bronchial occlusion in a COVID‐19 patient

**DOI:** 10.1002/ccr3.6754

**Published:** 2022-12-21

**Authors:** Satoshi Tanaka, Yoshihiro Takayama, Riiko Kitou, Ryo Asakawa, Satoshi Tobita, Akihiro Ike, Masahiro Kawada, Suguru Yamamoto, Kiyonobu Ueno

**Affiliations:** ^1^ Department of Respiratory Medicine Osaka General Medical Center Osaka Japan; ^2^ Department of Thoracic Surgery Osaka General Medical Center Osaka Japan; ^3^ Division of Trauma and Surgical Critical Care Osaka General Medical Center Osaka Japan

**Keywords:** bilateral pneumothorax, bronchial occlusion, chest drainage, COVID‐19, pleurodesis

## Abstract

Coronavirus disease 2019 (COVID‐19) has become a worldwide outbreak, and it can cause various symptoms and complications. However, pneumothorax secondary to COVID‐19 is relatively uncommon. We herein report a 60‐year‐old man with bilateral refractory pneumothorax with severe COVID‐19. In patients with poor general health and who are difficult to undergo surgery for pneumothorax post‐COVID‐19, internal treatments such as chest drainage, bronchial occlusion, and pleurodesis are essential to relieving refractory pneumothorax. It also indicates that autologous blood patch pleurodesis is a useful method in terms of efficacy and side effects.

## Abstract 2

Bilateral pneumothorax secondary to COVID‐19 is a very rare complication and can be refractory. In patients with poor general condition and who are unfit for surgery, combining internal treatments (chest drainage, pleurodesis, and bronchial occlusion) is essential for relieving refractory pneumothorax.

## 1 INTRODUCTION

The outbreak of coronavirus disease 2019 (COVID‐19) infection has been widespread all over the world. COVID‐19 infection can cause various symptoms and complications^1^ and pneumothorax secondary to COVID‐19 is relatively rare. Pneumothorax as a complication of COVID‐19 infection occurs in 1% of hospitalized COVID‐19 patients^2^ and 2% of COVID‐19 patients in the intensive care unit.^3^ Simultaneous bilateral spontaneous pneumothorax is an extremely rare condition that is found in only 1% of all patients with spontaneous pneumothorax.^4^ There have been few reports of bilateral pneumothorax due to COVID‐19. There is no treatment strategy for secondary spontaneous pneumothorax due to COVID‐19. In patients with poor general condition and who are not fit for surgery, refractory pneumothorax must be cured by internal therapies (chest drainage, bronchial occlusion and pleurodesis, and so on). There are various drugs in pleurodesis and autologous blood is a better adhesion in terms of efficacy and side effects. We herein report a rare case of a severe COVID‐19 patient with bilateral refractory pneumothorax treated by combining internal treatments.

## 2 CASE HISTORY

**FIGURE 1:**
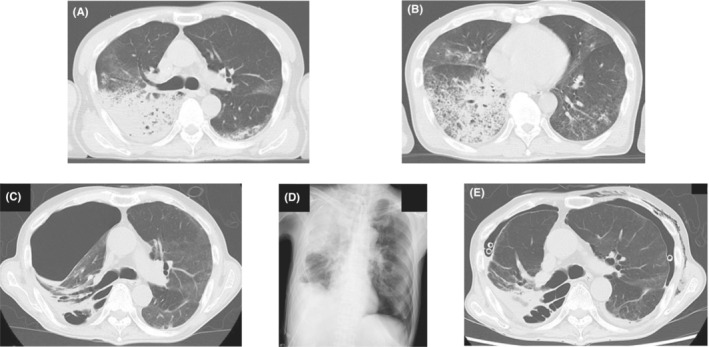
(A, B) Contrast computed tomography on admission shows infiltration shadows on both sides. (C) Computed tomography shows second right‐sided pneumothorax. (D) Chest X‐ray shows first left‐sided pneumothorax. (E) Computed tomography shows bilateral pneumothorax after bilateral tube drainage.

**FIGURE 2:**
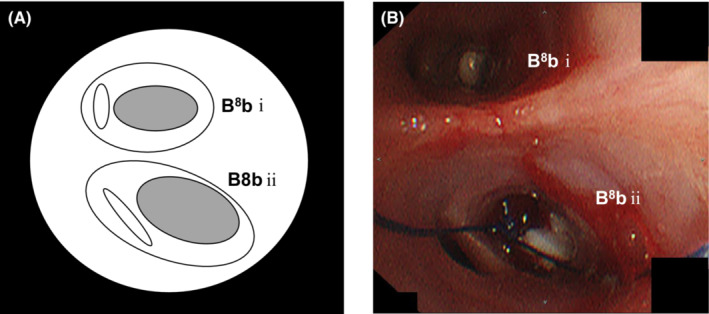
(A) Scheme of B^8^b on the left. (B) Flexible bronchoscopy shows bronchial obstruction using EWS® (a 6‐mm EWS® into B^8^bi and a 7‐mm EWS® into B^8^bii on the left).

A 60‐year‐old Japanese man with no medical, family, or smoking history was diagnosed with coronavirus disease 2019 (COVID‐19). Two weeks later, he was bought to our hospital in an ambulance because of high fever and dyspnea. He required oxygen supplementation at 15 L/min via an oxygen mask with an oxygen reservoir and he was immediately intubated. Computed tomography (CT) revealed bilateral diffuse infiltrations (Figure 1A,B) and he was diagnosed as having bacterial and secondary organizing pneumonia following COVID‐19 infection. He was treated with antibiotics, methylprednisolone, and heparin sodium. His respiratory condition improved gradually, and mechanical ventilation was discontinued on day 15. He complained of right chest pain on day 22, and CT revealed right‐sided pneumothorax. A chest drain was inserted immediately, which was removed on day 29 after pleurodesis with minocycline (MINO) 200 mg and OK‐432 5KE. However, the right‐sided pneumothorax recurred on day 50 (Figure 1C), and a left‐sided pneumothorax appeared on day 56 (Figure 1D). Chest drainage was performed on both sides, and an additional drain was placed on the right side (Figure 1E). He underwent pleurodesis on both sides (right side: twice with MINO 200 mg and OK‐432 5KE and twice with 100 ml of autologous blood, left side: twice with 100 ml of autologous blood); however, the air leak persisted. He was bedridden with advanced ICU‐acquired weakness and surgeons deemed him unfit for surgery. There were more leaks on the left side than on the right side; therefore, we performed a bronchial occlusion procedure using Endobronchial Watanabe Spigots® (EWS®; Novatech) for the left‐sided pneumothorax on day 68. We inserted a 6‐mm EWS® into B^8^bi and a 7‐mm EWS® into B^8^bii on the left (Figure 2A,B). Bronchial occlusion procedure was performed using flexible bronchoscopes (1T290 and P290, Olympus) and curette (CC‐4CR‐1, Olympus). The right lung expanded fully after bronchial occlusion and pleurodesis with 100 ml of autologous blood added on both sides, performed once on the right side and twice on the left side. Thereafter the air leaks on both sides stopped, and bilateral pneumothorax improved. The right drain was removed on day 73 and the left drain on day 75. Thereafter, there was no recurrence of pneumothorax and he was discharged on day 202 after rehabilitation for ICU‐acquired weakness.

## 3 DISCUSSION

Bilateral pneumothorax due to COVID‐19 has not been widely reported in the literature so far. The pathophysiology for the development of pneumothorax in patients with COVID‐19 is unclear; however, cystic and fibrotic structural changes caused by COVID‐19 may be related.^5^ In addition to this, treatment with positive pressure by non‐invasive or mechanical ventilation in patients with severe COVID‐19 infection may contribute to the development of pneumothorax.^6^ Systemic corticosteroids exhibit anti‐inflammatory and anti‐fibrotic effects in patients with severe COVID‐19 infection. However, corticosteroid therapy may also cause a delay in the wound‐healing process of pneumothorax.^7^ There is no established treatment strategy for secondary spontaneous pneumothorax due to COVID‐19. Surgical intervention presents a better outcome than tube thoracostomy in simultaneous bilateral primary spontaneous pneumothorax.^8^ However, in some patients surgical intervention for pneumothorax may pose a high risk from the perspective of general and respiratory conditions. In patients with poor general conditions and who are not fit for surgery, refractory pneumothorax must be cured by other internal therapies. Internal treatments for pneumothorax include procedures such as chest drainage, pleurodesis, and endobronchial approach.^9^ In some cases, it is important to combine multiple methods. We decided that our patient's general condition was not good enough to operate. Thus, the combination of bronchial occlusion and pleurodesis relieved the patient's bilateral refractory pneumothorax. Bronchial occlusion using an EWS® is one of the most effective and minimally invasive treatments for secondary refractory pneumothorax, postoperative pulmonary fistula, fistulous empyema, and bronchial fistula in patients with poor general condition. Bronchial occlusion alone or in combination with pleurodesis for secondary refractory pneumothorax, postoperative pulmonary fistula, or fistulous empyema enabled drain removal in 57.1%–86.0% of patients.^10^, ^11^, ^12^

Mitsuyama et al.^13^ reported that autologous blood patch pleurodesis may be a treatment option for recurrent and refractory pneumothorax secondary to COVID‐19. In our case, pleurodesis with MINO and OK‐432 was ineffective, whereas the one with autologous blood was effective. Pleurodesis with autologous blood has fewer side effects than that with other drugs such as OK‐432, talc, glucose, and minocycline hydrochloride^14^ Therefore, pleurodesis with autologous blood is a better strategy for refractory pneumothorax in patients with poor general condition after severe COVID‐19 infection. In the case of inoperable patients with refractory pneumothorax after severe COVID‐19 infection, bronchial occlusion, and pleurodesis can be key treatment strategies.

## 4 CONCLUSION

Bilateral pneumothorax can occur as a complication of COVID‐19 infection. Refractory pneumothorax in severe COVID‐19 patients with poor general conditions may be inoperable. In such situations, combining internal treatments such as chest drainage, bronchial occlusion, and pleurodesis is essential for relieving refractory pneumothorax.

## AUTHOR CONTRIBUTIONS

**Satoshi Tanaka:** Writing – original draft. **Yoshihiro Takayama:** Writing – review and editing. **Riiko Kitou:** Writing – review and editing. **Ryo Asakawa:** Writing – review and editing. **Satoshi Tobita:** Writing – review and editing. **Akihiro Ike:** Writing – review and editing. **Masahiro Kawada:** Writing – review and editing. **Suguru Yamamoto:** Writing – review and editing. **Kiyonobu Ueno:** Writing – review and editing.

## FUNDING INFORMATION

None.

## CONFLICT OF INTEREST

The authors have no conflicts of interest to disclose.

## ETHICAL APPROVAL

The patient was informed of this case report and a written consent form was obtained.

## CONSENT

Written informed consent was obtained from the patient to publish this report in accordance with the journal's patient consent policy.

## Data Availability

Data sharing is not applicable to this article as no new data were created or analyzed in this study.
